# Hepatocellular carcinoma with duodenal invasion resected subsequent to multimodal therapies: A case report

**DOI:** 10.1016/j.ijscr.2019.06.046

**Published:** 2019-06-26

**Authors:** Takashi Ito, Tetsuro Hirose, Atsushi Matsumoto, Akitada Yogo, Tomoko Okuno, Ryuichiro Doi

**Affiliations:** a Department of Surgery, Otsu Red Cross Hospital, Nagara 1-1-35, Otsu, Shiga, 520-8511, Japan; b Department of Gastroenterology, Otsu Red Cross Hospital, Nagara 1-1-35, Otsu, Shiga, 520-8511, Japan; c Department of Pathology, Otsu Red Cross Hospital, Nagara 1-1-35, Otsu, Shiga, 520-8511, Japan

**Keywords:** HCC, hepatocellular carcinoma, TACE, transarterial chemoembolization, GI, gastrointestinal, HPPD, hepatectomy with pancreas-preserving partial duodenectomy, JSH, the Japan Society of Hepatology, BCLC, Barcelona Clinic Liver Cancer, PIVKA-2, protein induced by vitamin K absence or antagonist -Ⅱ, AFP, α-fetoprotein, HPD, hepatectomy with pancreaticoduodenectomy, DEB-TACE, drug-eluting beads-transarterial chemoembolization, Hepatocellular carcinoma, Hepatectomy, Pancreas-preserving partial duodenectomy, Duodenal invasion, Sorafenib, Case report

## Abstract

**Introduction:**

Gastrointestinal (GI) involvement in hepatocellular carcinoma (HCC) is uncommon. In particular, HCC with duodenal invasion is known to be a rare condition. In such cases, surgical indication has been generally negative except in few reported cases. To our knowledge, this report describes the first case of HCC with duodenal invasion, resected by hepatectomy accompanied by pancreas-preserving partial duodenectomy (HPPD) following multimodal therapies including systemic sorafenib administration.

**Case presentation:**

A 65-year-old man had been repeatedly treated for multiple HCCs by transarterial chemoembolization (TACE) and sorafenib. However, the main tumor formerly ruptured began to involve his duodenum, causing GI bleeding. The collateral vessels from the pancreatic and omental branches entered the tumor and nullified the transarterial hemostatic embolization. Hence, HPPD was performed to preserve the major Vater papilla. Histopathological examination revealed poorly-to -moderately differentiated HCC cells invading the duodenum.

**Discussion and conclusion:**

HPPD treatment successfully removed HCC with duodenal invasion achieving viable tumor clearance status (R0). We underline the importance of achieving viable tumor clearance status at any time during the treatment course of patients with advanced HCC as this approach may be the only approach to enable HCC patients with duodenal invasion to resume a healthy life.

## Highlights

• Hepatocellular carcinoma (HCC) with duodenal invasion is a very rare occurrence.
• Aggravated disease condition of HCC with duodenal invasion usually makes surgical treatment not advisable.
• We present the first case of HCC with duodenal invasion resected following a multimodal therapy that included sorafenib.
• Partial hepatectomy in conjunction with partial duodenectomy was selected to lessen surgical stress.
• Surgical resection should be taken into consideration as a therapeutic choice even in progressive HCC disease condition.

## 1 Introduction

Hepatocellular carcinoma (HCC) is a type of primary liver tumor which may result from chronic hepatitis or liver cirrhosis [[1], [2], [3]]. There have been guidelines, both within and outside Japan, for the treatment of HCC [4,5] which limit surgical indication on the basis of the number of tumors and the liver capacity. There are several surgical case reports presenting cases of multiple HCCs, advanced locally, or presenting invasion of other organs [[6], [7], [8], [9]]. However, the surgical approach has been limited by the treatment guidelines for HCC in favor of other therapeutic options such as transarterial chemoembolization (TACE) or chemotherapy. As a consequence of this, a lower number of surgical case reports for HCC has been reported.

In this case report, we performed surgery on a patient with multiple HCCs, one of which involved the duodenum. The onset of disease was marked by the rupture of the main tumor in segment 5 which had been initially controlled by repetitive TACE along with 3 other lesions which were subsequently detected around the main tumor. While the other lesions were kept under control by TACE, the lesion in segment 5 proved to be resistant to TACE and chemotherapy, gradually developing and invading the duodenum, inducing gastrointestinal (GI) bleeding. The transarterial hemostatic embolization was disturbed due to the presence of collateral vessels. Hence, the liver tumor involving the duodenum was removed en bloc by hepatectomy accompanied by pancreas-preserving partial duodenectomy (HPPD). After 36 months from the surgery, the patient is still alive with no viable lesion, living a disease-controlled life.

The reference therapeutic guideline for the treatment of HCC in Japan is the Japanese Society of Hepatology (JSH) HCC Guidelines 2017. These guidelines provide an algorithm for HCC treatment [4]. The algorithm is based on liver function, tumor size and number and share some similarities with the Barcelona Clinic Liver Cancer (BCLC) staging [5], although more patients could be candidates for hepatectomy on JSH algorithm. According to the algorithm, surgery was not indicated for our patient, as he presented 4 or more HCC lesions. Moreover, there is no definite recommendation for secondary and tertiary treatments against the residual lesions if the other recommended type of therapies failed. However, in some selected cases, multimodal HCC therapy followed by surgery may enable the patient to live a disease-controlled life even after the failure of TACE and pharmacotherapy. We report here one of such cases. This work has been reported in line with the SCARE criteria [10].

## 2 Case presentation

**Fig. 1:**
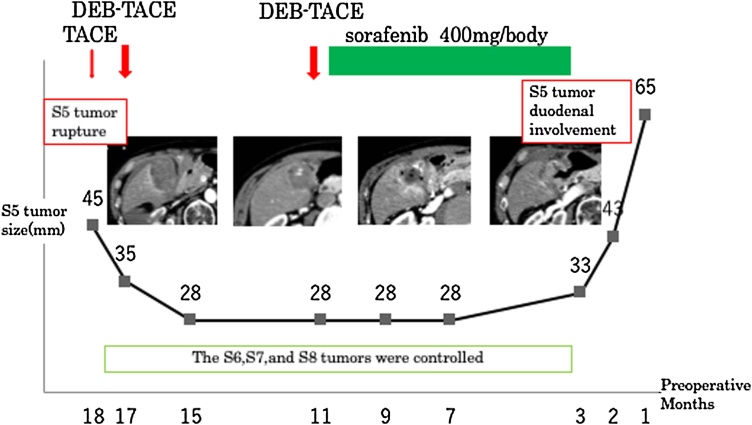
The patient’s preoperative course: He had been initially treated with emergent arterial embolization of the ruptured main tumor in the segment 5. Multiple HCC lesions were treated by consecutive TACE and sorafenib administration. Although, other small HCC lesions remained controlled, the main pre-ruptured tumor in the segment 5 developed up to 6.5 cm in diameter directly involving the first and second portion of the duodenum.

A 65-year-old male positive for hepatitis C virus antibody had been treated for HCCs in the liver segments 5, 6, 7, 8 in our hospital since October 2014. The patient was initially treated for the ruptured HCC in the segment 5 with TACE, while subsequent examinations detected other HCCs in segments 6, 7 and 8 which were similarly treated by 2nd line TACE, following the Japanese HCC therapeutic guidelines. After the treatment, the tumors in segments 6 and 7 were well controlled, while the pre-ruptured tumor in segment 5 and the lesion in segment 8 still remained viable. Hence, a 3rd line TACE was performed after 6 months: while the segment 8 tumor were well embolized after the treatment, the segment 5 tumor could not be embolized completely. Subsequently, it continued to gradually develop, as the caudal side of this tumor was perfused by the gastroepiploic artery, due to the ruptured tumor progression into the greater omentum. The collateral vessels from the omental branch into the tumor disturbed further TACE, as we suspected it could induce ischemia in the GI tract. Hence, the chemotherapeutical sorafenib was selected and administered repeatedly (Fig. 1).

Since this pre-ruptured tumor began and continued to involve the duodenum in the next six months, causing anemia and malnutrition, even further administration of sorafenib became difficult. At this point, the patient was referred to our surgical department for the first time. His critical situation compelled us to consider a surgical intervention.

**Fig. 2:**
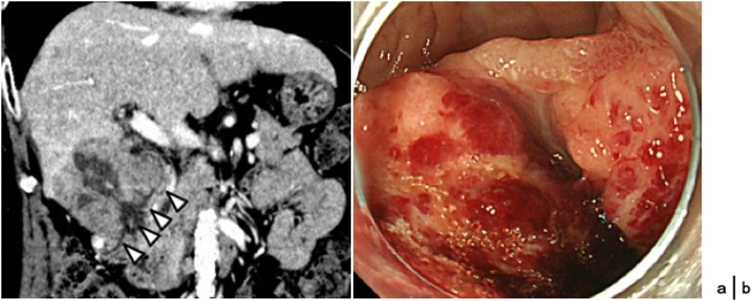
(a) Abdominal enhanced CT scan showed the tumor in the liver segment 5, invading the first and second portion of the duodenum (arrowheads). (b) An upper GI endoscopy revealed an ulcerative lesion on the posterior wall of the duodenal bulb.

A pre-operative examination by CT revealed a massive HCC in segment 5 of 6.5 cm in diameter, protruding from the liver and penetrating the duodenal wall (Fig. 2a), while other small HCC lesions appeared to be controlled. A gastroendoscopy revealed an ulcerative mass lesion in the posterior wall of the duodenal bulb (Fig. 2b), which was revealed to be HCC following histology analysis of a biopsy sample. Laboratory analysis revealed anemia (hemoglobin 9.2 mg/dl) and low albumin level (3.0 g/dl), while PIVKA-2 and α-fetoprotein levels were extremely high (2560 mAU/ml and 13,3000 ng/ml, respectively). The retention rate for indocyanine green until 15 min was 8.5%, revealing that his liver function was not compromised. During the preoperative evaluation, repeated blood transfusions became necessary, while communicating collateral vessels from the pancreatic and omental branch into the tumor disturbed hemostatic embolization. Since the disease condition aggravated by the tumor progression became a life-threatening oncological emergency, we were compelled to operate on the patient. However, considering his anemic and malnutritional preoperative conditions, we decided to exclude highly invasive surgical approaches such as hepatectomy with pancreaticoduodenectomy (HPD), and instead attempted to do HPPD to lessen surgical stress.

**Fig. 3:**
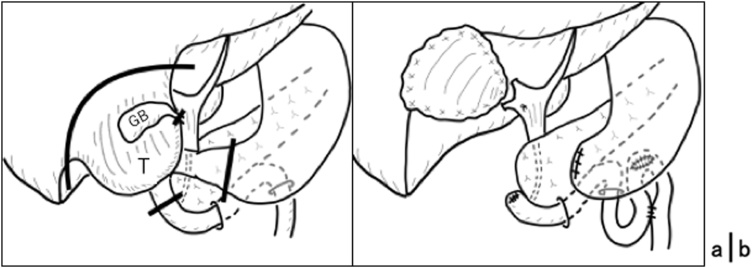
The schemas of HPPD. (a) The dissected points are shown by heavy lines. T: tumor, GB: gall bladder. (b) The way of reconstruction is shown.

During surgery, we found that the ruptured HCC in segment 5 further developed to became about 10 cm in diameter, involving the duodenal wall, fortunately without any macroscopic peritoneal dissemination. This corresponded to a grade evaluation of S2 (invasion to other organs) and P0 (no peritoneal dissemination) according to the General Rules for the Clinical and Pathological Study of Primary Liver Cancer 2019 by Liver Cancer Study Group of Japan [11]. We started the surgical treatment with the partial hepatectomy of segment 5 using the Pringle maneuver, and removed the tumor with the liver parenchyma as surgical margin (Fig. 3a). We did not perform anatomical resection of the segment 5 considering the past multiple HCCs status and also to avoid a major HPD in case we would be forced to add pancreaticoduodenectomy during the operation. The gall bladder was also removed as it was attached to the tumor. After the resection of the tumor from the liver, we inserted a thin Nelaton catheter from the cystic duct into the common bile duct to investigate the approximate location of the major papilla of Vater and its relation to the tumor. The inspection suggested the possibility of choosing HPPD and avoiding HPD, even after taking en bloc resection into consideration. On the basis of this observation, we subsequently dissected the first and second parts of the duodenum along the pancreas head and cut the minor pancreatic duct after its ligation. Then the duodenal inner lumen was opened to directly confirm the preservation of the major papilla of Vater. We removed the liver tumor and gallbladder en bloc with the first and second parts of the duodenum, finally transecting directly oral side to the papilla using a surgical stapling device. The reconstruction was performed using the Billroth II approach as in distal gastrectomy (Fig. 3b). Blood loss amounted to two liters due to the bleeding from the fragile collateral vessels surrounding the extended tumor and from those developed in the adhesional planes that formed due to the previous TACE treatments. We did not perform further peritoneal resection since no peritoneal disseminated lesion was found. Therefore, we evaluated the achieved resection equivalent to grade R0 (no viable tumor remaining).

**Fig. 4:**
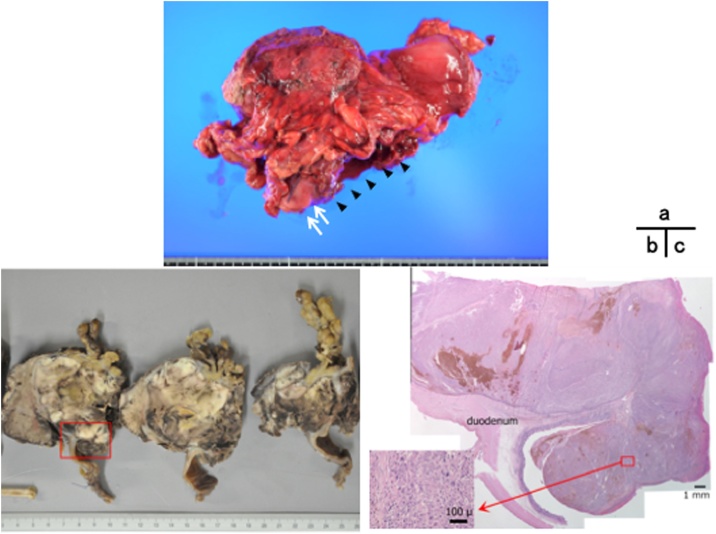
(a) The resected specimen. The tumor invaded the duodenum (arrows) and developed ulceration (arrowheads). (b) Macroscopically, the tumor was soft and the cut surface was grayish and brown. (c) Microscopic image of the boxed area. Duodenal invasion by the tumor cells was also microscopically evident (inset: high power view).

Post-operative course was uneventful except for a wound infection. He was discharged on the 45th postoperative day. Tumor markers were found to be in the normal range within three months after the surgery. Pathological examination was compatible with the pre-operative findings and revealed poorly-to-moderately differentiated HCC cells directly invading the duodenal wall and extending inside the duodenal lumen (Fig. 4). Approximately one quarter of the tumor was necrotic, demonstrating either the effects of repetitive TACE and the chemotherapy, although we cannot exclude spontaneous necrosis. Surgical margin was proved to be cancer negative.

After this surgery, the lesion in segment 8 remained stable. However, it was radio-frequency ablated twice as a preventive measure. Three years after the surgery the patient is still alive and leads a disease-controlled life.

## 3 Discussion

Extrahepatic metastases of HCC have been reported mainly in the lung and in the lymph nodes, while GI tract involvement in HCC is uncommon [[1], [2], [3]]. Its frequency has been reported as less than 1%, and among them, duodenal invasion is an extremely rare condition [12].

The first case of direct duodenal involvement in HCC was reported by Humbert et al. and caused upper GI bleeding [13]. Endoscopic findings of duodenal involvement or invasion in HCC have been usually reported to be either ulcerative lesions or submucosal tumors [12,14]. When we analyzed the literature reporting HCC cases with GI tract invasion leading to hemorrhage, we found several case reports in which patients were treated with transarterial embolization [15], endoscopic hemostasis, or ethanol injection [16]. However, in these cases, surgical indication was generally negative.

Surgical resection of HCC invading the duodenum has been rarely performed due to patient’s ill condition and due to surgical stress involving the necessity of maximal invasive HPD. Ja-Der Liang et al. reported 21 cases of HCC involving the duodenum following its invasion or due to the development of metastasis [8]. Among them, 50% of patients with duodenal invasion died within three months, reflecting their especially poor condition due to disease progression. Out of six patients who underwent surgery for duodenal involvement, only two were presented duodenal invasion.

Kato et al. presented surgical cases of HCC with GI tract involvement in their review paper [9]. According to their report, there are four treated cases of HCC with duodenal invasion reported to date. Among them, HPD was avoided in two cases, while in the other two HPD cases resulted in less than one-year survival, suggesting that the effect of high invasive surgery on patients in poor condition could lead to decreased survivability. This observation may support our less-invasive HPPD strategy. In addition, in all of the four reviewed cases, surgery was done without any type of pre-treatment, while in our case we utilized a combined TACE and sorafenib treatment before surgical resection. In our case, the effect of preceding therapies made the decision-making and surgery procedure more complex and difficult.

Sorafenib is a chemotherapeutical drug principally indicated for the treatment of patients with HCC without surgical indication, in agreement with the Japanese guidelines for HCC [4]. The response rate to this drug has been no more than 2–3% [17]. Hence, it is mostly used today as a palliative treatment rather than a curative one. Thus, systemic therapy by sorafenib has been generally indicated in patients with locally advanced or metastatic HCCs whose surgical treatment may not be expected. Retrospectively, we might have had a chance of surgical intervention when two of the first four tumors were controlled, but this happened prior to the patient’s introduction in our surgery department and prior to the repeated sorafenib administration. However, the initial number of tumors suggested against this decision in agreement with the therapeutic algorithm guidelines. To our knowledge, this case report describe the first successful resection of HCC with duodenal invasion following multimodal therapies including systemic sorafenib administration.

In this case report we present the possibility of a resection of the duodenal invasion of HCC by performing the HPPD procedure, which will surely relieve the surgical stress compared to the HPD procedure. Although the liver function in our patient allowed additional anatomical hepatectomy including right hemi-lobectomy, we chose to perform a partial liver resection to avoid a major HPD, since concomitant pancreaticoduodenectomy would have been required if we would have failed to preserve the major papilla near the tumor during the operation. Although we defined the resection corresponded to grade R0, our evaluation may be incorrect in precise as the tumor had previously ruptured and we suspected that remaining liver lesions could still be present. In support to our grade, however, during pre-operative CT and surgery we did not found any dissemination or other viable lesions. In conclusion, our available data demonstrate that the procedure achieved grade equivalent to R0 (all viable tumor has been removed). This result was achieved with minor surgical stress in comparison to major HPD resection.

Although the patient positively underwent additional radiofrequency ablation of segment 8 lesion twice after the surgery, this multimodal approach has further enabled him to live with no viable lesion to date for three years. Resection of the tumor even in oncological emergency might have helped him achieving a better prognosis in comparison to obstructed sorafenib administration that meant best supportive care.

## 4 Conclusions

We report a case of HCC with duodenal invasion successfully resected by HPPD following pre-treatment with repeated TACE and sorafenib administration. This result underlines the possibility of achieving a R0 (no viable tumor remaining) status at any time during treatment of patients with advanced HCC since this status may be the only way to enable patients with life-threatening disorders to resume a healthy life.

## Conflicts of interest

None.

## Funding

None.

## Ethical approval

Ethical approval was not required for this case.

## Consent

Written informed consent was obtained from the patient for publication of this case report and accompanying images. A copy of the written consent is available for review by the Editor-in-Chief of this journal on request.

## Authors’ contributions

TI, AY, and TH performed the operation.

AM managed the follow-up after operation.

TI and TH wrote the manuscript.

TO and RD revised the manuscript.

All authors read and approved the final manuscript.

## Registration of research studies

I have nothing to declare in this category.

## Guarantor

The guarantors of this study are TH and RD.

## Provenance and peer review

Not commissioned, externally peer-reviewed.
